# Anesthetic Management of a Pregnant Patient With Mixed Aortic Valve Disease Undergoing Cesarean Section

**DOI:** 10.7759/cureus.80670

**Published:** 2025-03-16

**Authors:** Jose Gerardo Lopez Saenz, Maria Fernanda Murillo Murillo, Shirley Acuna Chinchilla

**Affiliations:** 1 Anesthesiology, Hospital Mexico, San Jose, CRI; 2 Anesthesiology, Hospital San Rafael, Alajuela, CRI

**Keywords:** aortic valve regurgitation, aortic valve stenosis, cesarean section, combined spinal–epidural, obstetric anesthesia

## Abstract

Aortic stenosis is a condition with multiple etiologies that can remain clinically silent for years. The enhanced cardiovascular demands of pregnancy can unmask a previously undetected disease or worsen existing symptoms. Managing anesthesia in pregnant patients with cardiac valvular disease is a complex task that requires a coordinated, multidisciplinary approach.

This case report presents a 28-year-old pregnant Hispanic patient with known severe aortic stenosis who was admitted to our hospital at 30 weeks of gestation for multidisciplinary management and delivery planning. Her only symptom was dyspnea, classified as NYHA class II. Upon admission, a transesophageal echocardiogram (TEE) revealed severe subvalvular aortic stenosis caused by a membrane and moderate aortic valve regurgitation with a preserved left ventricular ejection fraction.

Initially, a cesarean section was planned due to breech presentation. The procedure was scheduled for 34 weeks but postponed to 36 weeks of gestational age due to the patient’s stable hemodynamic status. The anesthetic approach was a combined spinal-epidural technique that provided better hemodynamic stability and effective pain control and minimized the risk of broncho-aspiration and the maternal-fetal transfer of obstetric drugs associated with general anesthesia. Careful patient selection and continuous hemodynamic monitoring were essential to avoid complications and guarantee a successful outcome. This approach proved to be safe and effective for our patient.

This case highlights the importance of a multidisciplinary team approach in managing pregnant patients with cardiac disease. It also demonstrated that combined spinal-epidural anesthesia is a safe and viable option for anesthesiologists caring for these patients.

## Introduction

Cardiovascular disease affects between 1% and 4% of all pregnancies and remains the leading cause of maternal mortality in the United States, accounting for approximately one-third of all maternal deaths. Pregnant patients with aortic stenosis and New York Heart Association (NYHA) class III or IV have a peripartum morbidity rate of 10%, significantly higher than the 0.4% observed in those with NYHA class I or II. Aortic valve disease, though rare, is reported in 1.8 per 10,000 pregnancies in the United States [1-3].

Anesthetic management in pregnant patients with cardiac valvular disease is complex and challenging due to the physiological changes associated with pregnancy. These expected changes include increased cardiac output because of a higher heart rate and stroke volume, increased circulating blood volume, and decreased systemic vascular resistance. These changes can mimic symptoms of heart disease, leading to confusion and complicating diagnosis and management. As pregnancy progresses, the cardiovascular system undergoes significant strain to support fetal development, which can worsen preexisting cardiac conditions and unmask previously subclinical disease [3-6].

Patients with a fixed stroke volume due to an outflow tract obstruction face challenges in increasing it during pregnancy, which places them at a higher risk for cardiovascular complications both during pregnancy and delivery. Factors such as uterine contractions, pain, blood loss, inferior vena cava compression, uterine involution, and the hemodynamic effects of anesthesia can precipitate abrupt changes in cardiovascular function. These shifts can lead to acute decompensation [5,7].

Decisions regarding the birth should be made prior to the delivery date by a multidisciplinary team; in order to minimize complications. Cesarean delivery is generally reserved for obstetric indications or patients at high risk of hemodynamic instability during labor. The mode of delivery and anesthetic technique must be carefully individualized to optimize maternal and fetal outcomes [2].

Our case report describes a pregnant patient with severe aortic stenosis and moderate aortic regurgitation who faced successful cesarean delivery under combined spinal-epidural anesthesia, supported by intensive hemodynamic monitoring. This approach highlights the critical role of multidisciplinary collaboration and patient-specific anesthetic strategies in managing high-risk pregnancies complicated by significant cardiac disease.

## Case presentation

A 28-year-old Hispanic primigravida woman, 68.5 kg, 157 cm (body mass index: 27.8 kg/m²), was referred to the Department of Gynecology and Obstetrics at our hospital at 30 weeks and one day of gestation. Her medical history included iron deficiency anemia secondary to uterine myomas and a double aortic valvular lesion-severe aortic stenosis and moderate aortic regurgitation-diagnosed two years prior to her pregnancy. The underlying etiology of the aortic valve disease was attributed to a congenital bicuspid aortic valve.

The patient was asymptomatic but developed dyspnea with exertion during pregnancy, which led to her classification as NYHA class II. On physical examination, a grade III systolic heart murmur radiating to both carotid arteries was noted, along with mild (grade 1) edema in her lower extremities. Initial blood tests showed a hemoglobin level of 11.9 g/dL, hematocrit of 34.3%, platelet count of 209,000 cells/mm3, and no signs of abnormalities in hepatic or renal function (Table 1). Given her complex cardiac condition, the patient was admitted to our hospital for further evaluation and management by a multidisciplinary team.

**Table 1:** Patient's laboratory findings at admission. BUN: blood urea nitrogen, ALT: alanine transaminase, AST: aspartate transaminase, GGT: gamma-glutamyl transferase, NT-proBNP: N-terminal prohormone of brain natriuretic peptide.

Variable	Admission	Reference range
Hemoglobin (g/dL)	11.9	12.5-15.3
Hematocrit (%)	34.3	36-48
Platelets (cells/mm^3^)	209,000	150,000-445,000
Leukocytes (cells/mm^3^)	11,900	4,000-12,000
Neutrophils (cells/mm^3^)	8,925	1,600-6,000
Lactate dehydrogenase (IU/L)	170	205-350
Creatinine (mg/dL)	0.44	0.6-1.2
BUN (mg/dL)	5.7	8-25
Albumin (g/dL)	3.2	3.5-5.1
ALT (IU/L)	10	10-40
AST (IU/L)	17	10-42
Alkaline phosphatase (IU/L)	89	32-92
GGT (IU/L)	15	8-64
Total bilirubin (mg/dL)	0.32	0.5-1.5
Indirect bilirubin (mg/dL)	0.21	0.3-0.6
NT-proBNP (pg/L)	33.30	<125

**Figure 1:**
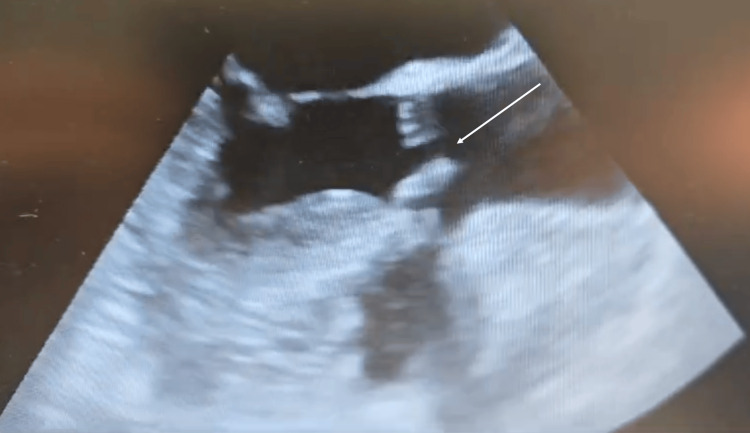
TTE parasternal long-axis view revealing severe aortic stenosis TTE: Transthoracic echocardiography

**Figure 2:**
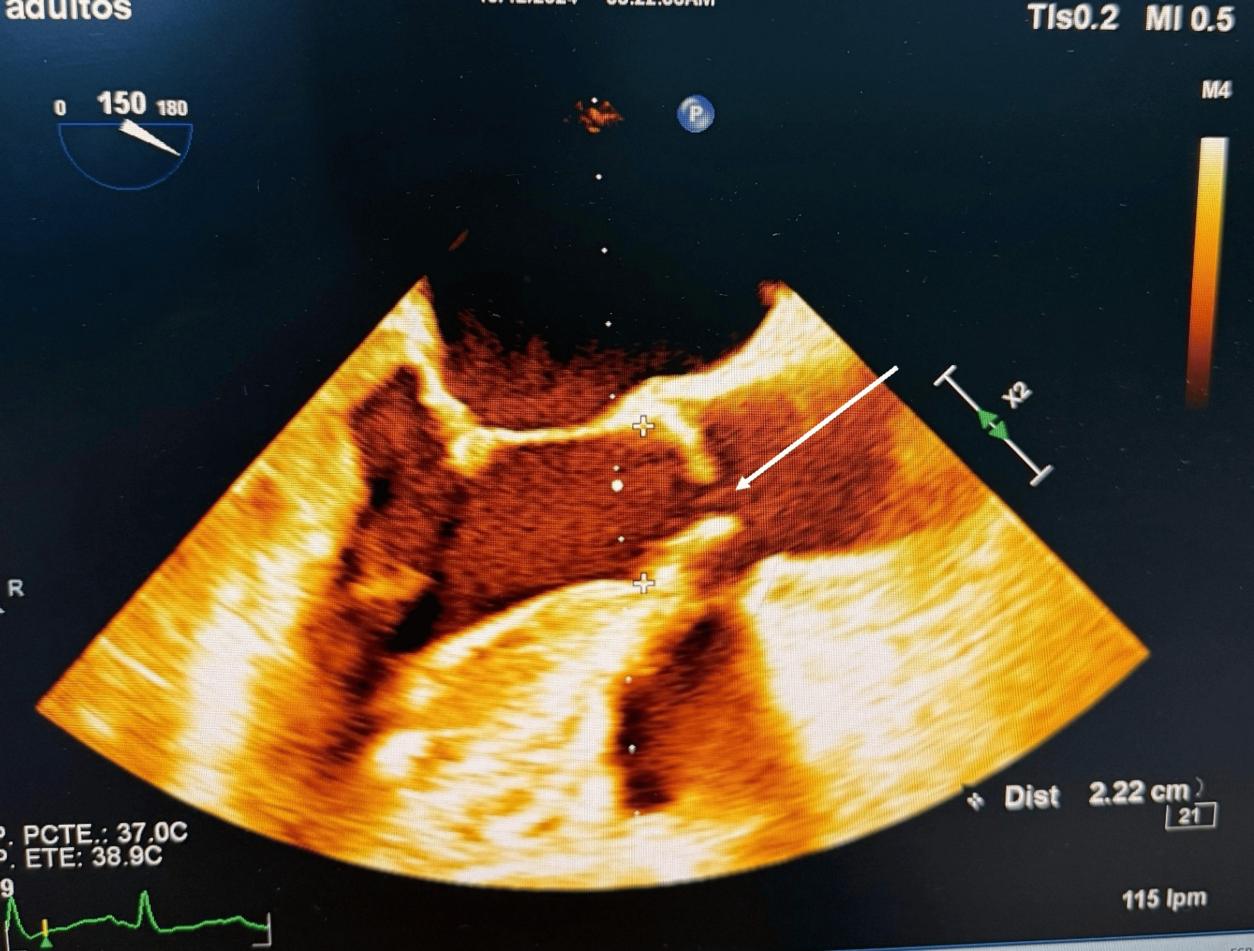
TEE mid-esophageal aortic valve long-axis view revealing severe aortic stenosis TEE: Transesophageal echocardiography

A transthoracic echocardiogram (TTE) revealed severe subvalvular aortic stenosis and moderate aortic valve regurgitation. These findings were confirmed by transesophageal echocardiography (TEE) (Figure 1 and Figure 2). 

Table 2 shows the comparisons in TTE and TEE findings.

**Table 2:** Echocardiographic measurements at admission. TTE: transthoracic echocardiogram, TEE: transesophageal echocardiogram, PSAP: pulmonary artery systolic pressure, EF: ejection fraction.

Measurements	TTE	TEE
Aortic valve	Severe subvalvular aortic stenosis due to a membrane. Tricuspid, thickened edges and restricted opening predominantly affecting the non-coronary cusp. Peak velocity 4.89 m/s, peak gradient 95 mmHg, mean gradient 58 mmHg, acceleration time 119 ms, dimensionless index 0.19, pressure half time 357 ms, aortic valve area 0.42 cm2 (0.27 cm2/m2 indexed)	Severe subvalvular aortic stenosis due to a membrane. Moderate aortic valve regurgitation secondary to jet from the subaortic membrane in contact with the left coronary cusp (4 mm prolapse). Pressure half time 349 ms, peak velocity 5.38 m/s, mean velocity 3.88 m/s, peak gradient 116 mmHg, mean gradient 71 mmHg
Tricuspid valve	Mild tricuspid insufficiency. Peak gradient 29 mmHg. Peak PSAP 29 mmHg. No pulmonary hypertension	Mild tricuspid insufficiency. Peak gradient 44 mmHg
Pulmonic valve		Mild pulmonic valve regurgitation. Diastolic peak gradient 18 mmHg
Left ventricle	EF 65%. Severe concentric hypertrophy. Global and segmental biventricular contractility preserved. Normal diastolic function with increased filling pressures. No intracavitary thrombus	Global and segmental biventricular contractility preserved. Severe concentric hypertrophy
Left atrium	Moderate dilation	Mild dilation
Right ventricle	Conserved EF. No intracavitary thrombus. No dilation	Conserved EF
Right atrium	No dilation	No dilation
Ascending aorta	The patient has a very small aorta and outflow tract, less than 2 cm on transthoracic echocardiography. For this reason, gradients and parameters may be higher. In the 2D views, the valve appears to open more, and by transthoracic 2D planimetry, it measures approximately 1-2 cm2 in area. Due to pressure recovery based on the measurements taken so far, the area would correspond to 0.5 cm2. However, the patient is pregnant, which significantly modifies her functioning, in addition to her small structures.	Aortic coarctation. Small-caliber aorta
Pericardium	No pericardial effusion	
Inferior vena cava	Diameter of 19 mm and collapse less than 50% on inspiration	

An obstetric ultrasound revealed a fetus in breech presentation, a normally positioned placenta, and normal amniotic fluid levels with an amniotic fluid index of 188 mm. The estimated fetal weight was 1977 grams, corresponding to the 58th percentile for gestational age.

Following a comprehensive multidisciplinary team meeting involving the obstetrics, cardiology, anesthesia, and cardiothoracic surgery teams, it was decided to prolong the pregnancy until the 34th week of gestation due to the patient’s stable hemodynamic status and absence of clinical deterioration. In the event that the patient exhibited any clinical or laboratory signs of worsening before reaching 34 weeks, a cesarean section would be performed due to obstetric indications. The patient was deemed suitable for cesarean delivery, with no contraindications.

The patient didn’t require invasive monitoring during hospitalization. Routine blood tests, including NT-proBNP, creatinine, and blood urea nitrogen, were performed every three days and remained within normal limits. No additional medications were required for her chronic conditions. She completed four doses of 6 mg dexamethasone for fetal lung maturity.

**Figure 3:**
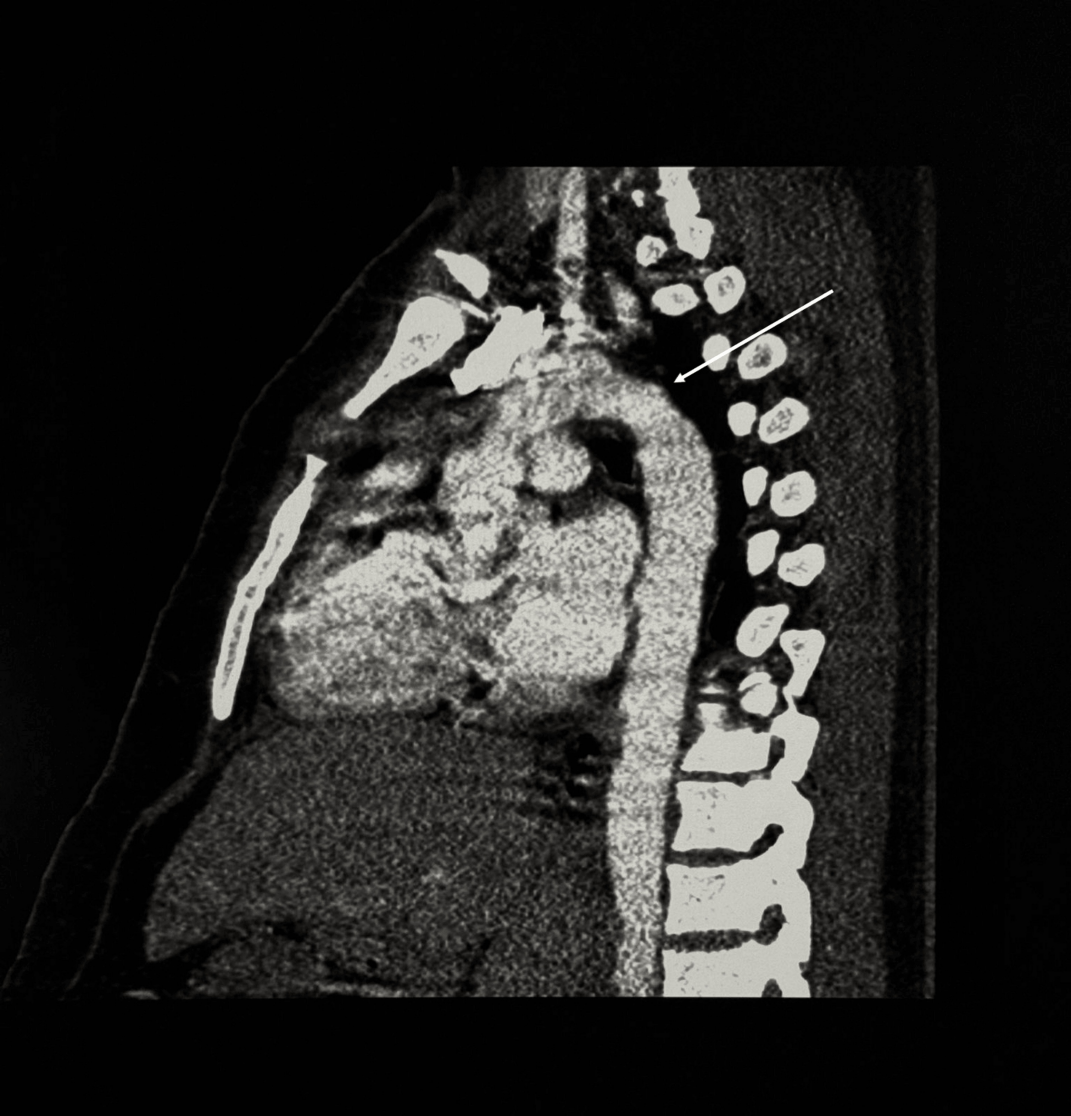
CT Angiography demonstrating normal aortic anatomy without evidence of coarctation. The image has artifacts because it is not a cardiac CT and lacks ECG synchronization. CT: Computed tomography; ECG: Electrocardiography.

Based on the TEE findings, a CT angiography was conducted to rule out aortic coarctation. Imaging revealed no evidence of coarctation in the thoracic aorta, with no dilation, constriction, or collateral circulation observed. The aortic arch appeared normal, and the relationship between the pulmonary artery trunk and the aorta was appropriate. Left ventricular hypertrophy with a wall thickness of 15 mm was reported (Figure 3).

In a subsequent multidisciplinary team meeting, it was determined that the pregnancy could safely be extended to 36 weeks of gestation, with weekly laboratory testing to ensure the patient’s stability. Cesarean section remained the planned mode of delivery based on the CT angiography results and the patient’s stable condition with preserved maternal-fetal well-being. A neonatal echocardiogram was also performed, revealing tricuspid valve dysplasia with regurgitation but no other abnormalities.

During the patient’s preoperative evaluation, no contraindications were found to spinal anesthesia. The patient remained asymptomatic, reported no dyspnea, and had a functional capacity exceeding four metabolic equivalents (METs). Her ASA status was classified as 3. Based on the patient’s clinical profile, the anesthetic plan was determined to be combined spinal-epidural anesthesia and invasive blood pressure monitoring using a ProAQT sensor. The cesarean section was categorized as NICE 4.

Before entering the operating room, the patient received 2 grams of intravenous ampicillin via an 18-gauge peripheral IV line. Standard monitoring was initiated upon arrival, including a three-lead electrocardiogram, pulse oximetry with an initial reading of 91%, and non-invasive blood pressure measurement, initially registered at 160/82 mmHg. A 20-gauge arterial cannula was inserted into the left radial artery, and invasive monitoring with a ProAQT sensor was initiated. During the placement of the arterial cannula, the patient had a transient peak of blood pressure of 170/84 mmHg, probably related to pain or light sedation, which was managed immediately with 1 mg of propranolol. Preoperative echocardiography in the operating room confirmed normal resting global left ventricular contractility. Supplemental oxygen at 4 L/min was delivered via a nasal cannula, and 50 mcg of fentanyl and 1.5 mg of midazolam were administered for sedation and analgesia.

Using spinal-epidural equipment, the epidural needle was advanced to the L2-L3 interspace. The epidural space was identified at a depth of 4.5 cm from the skin. A spinal needle was then introduced, and a bolus of 3 mg of 0.5% levobupivacaine and 20 mcg of fentanyl was administered intrathecally. Subsequently, an epidural catheter was placed, and a bolus of 100 mg of 2% lidocaine was delivered. A sensory block level of T4 was confirmed within minutes.

The patient remained hemodynamically stable throughout the procedure without requiring vasopressors or vasodilators, based on the information provided by the ProAQT sensor blood pressure monitoring. The neonate was delivered without complications, with APGAR scores of 7 and 8 at 1 and 5 minutes, respectively.

Following delivery, 3 units of oxytocin were administered as a slow IV bolus over 5 minutes to induce rapid uterine contraction and minimize cardiovascular effects. We opted for a slow bolus in our patient due to her normal resting heart contractility and normal hemodynamic parameters during surgery. Since the initial bolus did not achieve adequate uterine contraction, 3 additional units were required. The patient was hemodynamically stable throughout the procedure and had no cardiovascular effects after the oxytocin boluses. There was no excessive bleeding or other complications during the cesarean section.

An epidural bolus of 3 mg morphine and 5 mg of 0.25% levobupivacaine was administered, supplemented with 1 gm of IV acetaminophen for postoperative analgesia. In addition, a bilateral salpingectomy was also performed during the procedure. At the end of the surgery, the patient received an additional epidural bolus of 100 mg of 2% lidocaine.

**Figure 4:**
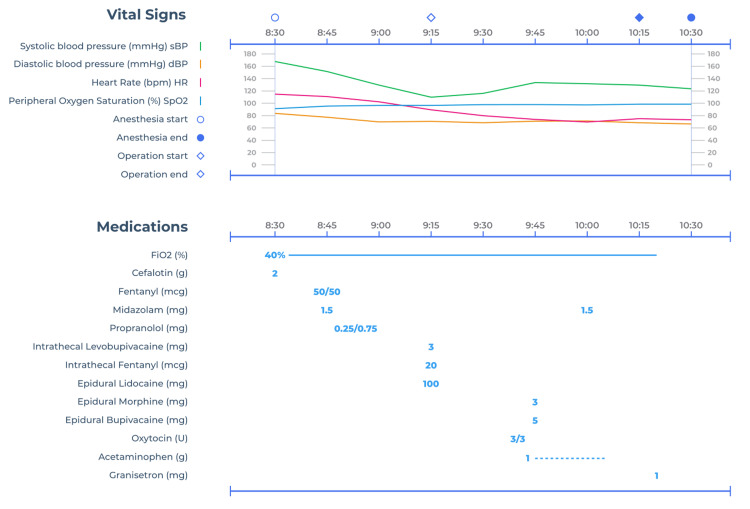
Patient’s intraoperative clinical course.

The surgery lasted 60 minutes, during which 500 mL of crystalloid fluids were administered. For postoperative pain management, an epidural elastomeric pump delivering 0.2% bupivacaine at 2 mL/hour for 50 hours was initiated, along with oral acetaminophen 1 gm every 8 hours. We opted for this analgesia regimen in our patient as our goal was to prevent pain, which could be associated with hemodynamic deterioration, while also improving adherence to the analgesic regimen and promoting a prompt recovery. No ultrasound-guided regional block was required for additional analgesia. The patient’s intraoperative clinical course and administered medications are summarized in Figure 4.

The patient was transferred to the intensive care unit for close monitoring. Postoperative neurological examination revealed paresthesia and reduced muscle strength in the left lower extremity, rating 2/5 distally and 3/5 proximally. After careful evaluation, it was determined that these symptoms were likely due to a slight dislodgement of the epidural catheter. Subsequently, the catheter was retracted by 2 cm. A follow-up neurological examination two hours later revealed a complete resolution of paresthesia and restoration of normal muscle strength. The epidural local anesthetic infusion was resumed, and the patient reported no pain on the visual analog scale (VAS).

On the first postoperative day, the patient reported moderate pain (VAS=4) at the incision site, particularly during movement. A bolus of 25 mg of 0.25% bupivacaine was administered and effectively alleviated the pain. The patient remained hemodynamically stable, with no recurrence of neurological symptoms. Twelve hours later, a 2 mg epidural morphine bolus was required for additional pain control. Postoperative laboratory tests were unremarkable except for mild anemia (hemoglobin of 11 g/dL).

On the morning of the second postoperative day, it was discovered that the epidural catheter was no longer in place. This was possibly due to being accidentally dislodged during the night. No active bleeding from the epidural catheter insertion site was found. Pain management was transitioned to intravenous metamizole and oral acetaminophen, which provided adequate relief. The patient’s clinical course remained uneventful, with no cardiac or pulmonary complications. Five days later, she was discharged from the hospital in good condition.

## Discussion

This case highlights the complexities of anesthetic management for a pregnant patient with severe subvalvular aortic stenosis, moderate aortic regurgitation, and iron deficiency anemia. It emphasizes the importance of close monitoring and specialized care in minimizing maternal and fetal risks. A multidisciplinary approach was essential to developing an optimized care plan and delivery strategy.

Valvular heart disease accounts for approximately 30% of all cardiac conditions complicating pregnancy [5]. Pregnant patients with heart disease are highly susceptible to adverse cardiovascular events, with an estimated 68% of pregnancy-related deaths due to cardiovascular conditions considered preventable [8].

Congenital bicuspid aortic valve, as seen in our patient, is the most common etiology of aortic stenosis in pregnancy. Severe aortic stenosis carries significant risks for both the mother and fetus, including pulmonary edema, arrhythmias, heart failure, and maternal death, as well as stillbirth, neonatal death, and preterm birth. However, maternal mortality and the need for aortic valve surgery during pregnancy remain low [6,9,10].

Pregnancy induces several physiological changes that can be seen during echocardiography. These changes include increased left ventricular mass due to reversible eccentric hypertrophy and enlargement of atrial and ventricular chambers. These alterations result from elevated blood volume and can eventually lead to annular dilation and increased transvalvular velocities. The transvalvular gradient may gradually exceed 50 mmHg as blood volume increases and systemic vascular resistance decreases. While tricuspid and pulmonic valve regurgitation is common in pregnancy, aortic regurgitation is not a typical finding [11,12]. Despite her mixed aortic valve disease, our patient’s condition remained stable throughout pregnancy.

Hemodynamic changes in pregnancy include increased cardiac output, plasma volume, heart rate, and red blood cell mass. As well as decreased systemic vascular resistance and hematocrit. Aortic stenosis primarily causes increased afterload, which leads to increased intracavitary pressure and wall stress. This can result in concentric hypertrophy, reduced diastolic compliance, and an imbalance between oxygen supply and demand, further impairing cardiac function. Hypovolemia, aortocaval compression in the supine position, and sympathetic blockade from neuraxial anesthesia can exacerbate the condition [11,13-15]. Anesthetic management in patients with aortic stenosis should focus on optimizing preload and cardiac output, avoiding heart rate extremes, preventing abrupt decreases in systemic vascular resistance, and maintaining sinus rhythm [12].

In our patient, the mode of delivery was determined by obstetrical indications during a multidisciplinary team discussion. We opted for combined spinal-epidural anesthesia for several reasons. The patient remained hemodynamically stable throughout her hospitalization, with no exacerbation of dyspnea. This indicates effective physiological compensation despite the increased demands of pregnancy. Echocardiographic imaging reported adequate contractility and ejection fraction. General anesthesia posed risks such as broncho-aspiration, thromboembolism, inadequate pain control, and neonatal depression due to maternal-fetal drug transfer. Spinal-epidural anesthesia enabled the use of low spinal doses to achieve a rapid onset, while epidural lidocaine was used to reach a T4 sensory level, eventually minimizing hemodynamic instability [16-18]. Additionally, having the option of postoperative pain control via the neuraxial route reduces abrupt hemodynamic changes caused by pain and facilitates early ambulation, thereby lowering the risk of thromboembolism.

The addition of opioids in spinal anesthesia enhanced intraoperative analgesia, while epidural morphine provided effective postoperative pain control without significant cardiovascular depression. Acetaminophen was used as part of a multimodal analgesic strategy. Although regional anesthetic blocks were an option, they were not required as pain was well-managed with epidural opioids in our patient [19].

The patient remained asymptomatic, and intraoperative transthoracic echocardiography showed no significant changes from prior evaluations. Invasive hemodynamic monitoring with a ProAQT sensor and PulsioFlex monitor provided real-time data that allowed rapid correction of any hemodynamic alterations. This system supported the safe administration of neuraxial anesthesia in severe aortic stenosis without significant hemodynamic instability [17]. Fluid status was optimized using parameters such as stroke volume variation, cardiac output, and cardiac index, all of which remained within normal limits during surgery. A beta-blocker was administered to address preoperative tachycardia, and low doses of midazolam and fentanyl were used to maintain cardiovascular stability.

There are several case reports on the use of combined spinal-epidural for cesarean section in patients with aortic stenosis. One involved a patient with aortic stenosis and 60% obstruction of the left anterior descending coronary artery. This patient experienced ST depression as a complication when the mean blood pressure dropped below 70 mmHg; however, there were no other complications, and the delivery outcomes were positive [20]. The other case report documented good hemodynamic stability, no complications, and effective pain management [21], while another case demonstrated that this technique was safe and effective [22].

This case highlights that using combined spinal-epidural anesthesia with meticulous hemodynamic monitoring provides a safe and effective strategy for managing pregnant patients with severe aortic stenosis. The importance of personalized care, multidisciplinary collaboration, and good hospital resources and supplies ensures optimal outcomes for both mother and baby.

## Conclusions

The successful management of a pregnant patient with severe subvalvular aortic stenosis and moderate aortic regurgitation emphasizes the importance of a multidisciplinary approach to achieving optimal outcomes for both the mother and the baby. The use of combined spinal-epidural anesthesia, carefully tailored to the patient’s unique hemodynamic needs and supported by continuous invasive monitoring, proved to be an effective technique for our patient. Close monitoring of her condition throughout the perioperative period played a critical role in preventing complications. This case highlights the importance of patient selection, personalized approaches, and the use of multimodal analgesia. All of these contributed to effective pain control and facilitated a smooth recovery for our patient.
